# The Association Between Job Stress and Intention to Leave in Healthcare Institutions: A Systematic Review and Meta-Analysis

**DOI:** 10.3390/healthcare14162541

**Published:** 2026-08-14

**Authors:** Enes Kaya, Nazmiye Ekinci, Imran Aslan, Juan Gómez-Salgado, Feten Fekih-Romdhane, Carlos Laranjeira, Murat Yıldırım

**Affiliations:** 1 Department of Healthcare Management, Kagizman School of Applied Sciences, Kafkas University, 36700 Kars, Türkiye; 2 Healthcare Management Department, Faculty of Health Sciences, Bingöl University, 12000 Bingöl, Türkiye; 3 Department of Sociology, Social Work and Public Health, Faculty of Labour Sciences, University of Huelva, 21071 Huelva, Spain; 4 Safety and Health Postgraduate Program, Universidad Espíritu Santo, Guayaquil 0901952, Ecuador; 5 Department of Psychiatry “Ibn Omrane”, The Tunisian Center of Early Intervention in Psychosis, Razi Hospital, Manouba 2010, Tunisia; 6 Faculty of Medicine of Tunis, Tunis El Manar University, Tunis 1007, Tunisia; 7 School of Health Sciences, Polytechnic University of Leiria, Campus 2, 2411-901 Leiria, Portugal; 8 Centre for Innovative Care and Health Technology (ciTechCare), Polytechnic University of Leiria, Campus 5, 2414-016 Leiria, Portugal; 9 Comprehensive Health Research Centre (CHRC), University of Évora, 7004-516 Évora, Portugal; 10 Department of Psychology, Faculty of Science and Letters, Agri Ibrahim Cecen University, 04100 Ağrı, Türkiye; 11 Psychology Research Center, Khazar University, AZ1096 Baku, Azerbaijan

**Keywords:** health, human resource management, job stress, turnover intention, healthcare institutions, meta-analysis

## Abstract

**Background:** Job stress is a pervasive issue in modern workplaces and is associated with numerous adverse outcomes, including employees’ turnover intention when stress levels are high. **Objective:** This study aimed to examine the association between job stress and turnover intention among employees working in healthcare institutions through meta-analysis. **Methods:** A systematic literature review and meta-analysis were performed following PRISMA guidelines. The study was registered in the International Prospective Register of Systematic Reviews (PROSPERO; ID: CRD42024581906). Considering variations in sample sizes, publication years, and measurement scales, a random-effects model was employed. Effect sizes were illustrated with a forest plot, and publication bias analyses were performed. Subgroup analyses were conducted by occupational group, COVID-19 period, and geographic region, while meta-regression tested the moderating effects of publication year. Study quality was assessed using the AXIS tool. **Results:** Based on predefined inclusion criteria, 38 independent studies were included. Findings revealed a moderate-to-high, positive, and statistically significant relationship (r = 0.441; 95% CI [0.389, 0.490]) between job stress and turnover intention among healthcare workers. Moreover, although between-study heterogeneity was substantial (I^2^ = 95.5%), the direction and statistical significance of the association remained consistent across different countries and healthcare systems. **Conclusions:** This meta-analysis demonstrated a statistically significant positive association between job stress and turnover intention among healthcare workers (r = 0.441). Although substantial heterogeneity was observed across studies, the relationship remained consistent across occupational groups, regions, and study contexts. Addressing job stress may therefore play an important role in supporting workforce retention in healthcare organizations.

## 1. Introduction

The quality of a company’s workforce greatly influences the organization’s overall performance. Employees with extensive experience, deep expertise, supportive knowledge, and strong skills become invaluable assets. Effective human resource management is crucial in this regard, as it enables organizations to harness employees’ potential for sustainable growth. Poor management, however, can reduce motivation, lower productivity, and even increase turnover [1].

Stress is an intense state of tension that exerts pressure on an individual’s emotional state, mental processes, and overall well-being [2]. When an employee is unable to cope with workplace demands, conflict may arise either among employees or between the employee and their work. Furthermore, such conflict often leads to personal dysfunction, manifesting in physiological and emotional symptoms within the workplace. This personal dysfunction is referred to as “job stress” [3]. Employees exposed to high levels of job stress and compelled to struggle with its consequences may eventually experience illness, burnout, and even become incapable of working. In many cases, this situation may lead employees to develop turnover intentions.

Today, turnover intention has become a significant issue, whether employees leave voluntarily or are dismissed by their employers. Nguyen et al. emphasize that high turnover rates result not only in substantial recruitment, training, and replacement costs for organizations but also in the loss of talent and corporate knowledge. Moreover, it has been found that high turnover rates negatively affect the motivation, engagement, and commitment of remaining employees, thereby diminishing company performance and productivity [4].

In recent years, job stress and turnover intention have become focal points of attention. Professions in the healthcare sector, such as nursing and medicine, are among the most stressful fields, and healthcare institutions are reported to have some of the highest employee turnover rates. Therefore, in this study, articles focusing on job stress and turnover intention within healthcare organizations have been systematically examined. Although the relationship between job stress and turnover intention has been widely documented in the organizational behavior and healthcare literature, existing evidence remains fragmented with respect to the quantitative magnitude of this association within healthcare settings and its consistency across professional and national contexts. Prior reviews have largely focused on narrative syntheses or context-specific findings, limiting their usefulness for strategic human resource management decisions.

The present study addresses this gap by providing a healthcare-specific meta-analytic synthesis that quantifies the pooled association size of job stress on turnover intention. Unlike prior reviews and meta-analyses conducted in general human resource management or mixed-sector samples, this study focuses exclusively on healthcare employees and systematically examines whether the magnitude of the relationship varies across professional groups, countries, cultural contexts, and time periods. By doing so, the study offers a more precise and contextually grounded understanding of job stress as a structural retention risk in healthcare organizations, thereby enhancing the relevance of meta-analytic evidence for strategic human resource management and workforce planning decisions in the healthcare sector.

## 2. Literature Review

### 2.1. Job Stress

Stress refers to an emotional state that arises when individuals perceive a situation’s demands as surpassing their coping abilities, thereby endangering their well-being [5]. Stress represents a state of tension that affects an individual’s emotions, cognitive processes, and overall condition [6]. According to Selye, the stress process begins with an alarm reaction to the situation, is followed by resistance to the stressor, and ultimately leads to an exhaustion phase [5].

Stress arises when an individual appraises the demands of an external situation as exceeding their perceived coping abilities. According to the stressor-strain perspective, job stressors serve as the stimuli that initiate this process, while symptoms such as tension, anxiety, and exhaustion represent its immediate outcomes [7]. Workplace stress has become a serious concern for corporate management today [6]. Job stress is described as an emotional or psychological state that emerges when an employee perceives that the demands of a workplace situation exceed their available resources and coping abilities [8].

Employees exposed to prolonged job stress may eventually become unable to perform their work. In more severe cases, stress can lead to illness or resignation [6]. Researchers have demonstrated that job stress significantly affects not only employees’ work lives but also their personal lives [8]. According to Handoko, job stress is a state of tension that influences an individual’s emotions, cognitive processes, and overall well-being [6]. Job stress is characterized as a condition that creates physical and psychological imbalance, generates tension, and affects individuals’ emotions, cognitive processes, and general mood [9].

Job stress arises from the mismatch between the goals or demands expected by the organization and the individual’s capacity to meet those expectations. When individuals psychologically and physically experience this mismatch, job stress emerges, impacting their emotions, thought processes, and overall mood [2]. Robbins et al. identify indicators of job stress as task demands, role demands, interpersonal pressures/strains, an unclear organizational structure, and disruptive forms of organizational leadership [9]. When individuals are unable to cope with job stress, deterioration may occur in their capacity to interact with their surroundings and perform their work [2]. Excessive stress can threaten an individual’s ability to manage their environment, leading to the development of various stress symptoms that may hinder job performance [6].

### 2.2. Turnover Intention

Turnover refers to the voluntary or involuntary permanent departure of an employee from an organization. High turnover rates can disrupt operations due to the loss of experienced personnel and can increase recruitment and training costs. Iqra et al. define turnover intention as the employee’s plan to leave their current job or the employer’s plan to terminate the employee [6]. When employees with turnover intentions act on their intentions, the employee turnover rate within the organization increases. Defined as the ratio of departures and arrivals within a given period to the total workforce, a high employee turnover rate imposes high costs on organizations related to new employee recruitment, training, and adaptation processes [10]. This situation negatively affects employee motivation and leads to a decline in job performance. Particularly, the departure of talented employees, who are considered human capital, can lead to an increased workload for managers and create planning difficulties [11].

Wibowo et al. outline several factors that may influence turnover intention. First, job satisfaction, career development opportunities, work environment and work–life balance can be associated with turnover intention. Second, salary dissatisfaction; inadequate compensation or lack of supportive benefits may drive individuals to leave an organization. Third, organizational commitment; employees’ loyalty to the company’s values, goals, and culture can affect their desire to stay. Employees who are emotionally attached to their organizations tend to have lower turnover intentions. Fourth, high workload and stress can lead to physical and mental exhaustion, prompting a desire for a more enjoyable or balanced career. Fifth, ineffective leadership and management; leadership styles that fail to provide clear guidance, support, and motivation may increase turnover intention [2,12]. The following section provides information on the relationship between job stress and turnover intention, which are the primary topics examined in this study.

### 2.3. The Relationship Between Job Stress and Turnover Intention

Today, as a result of various job demands, job stress has become a relevant and potentially significant problem affecting employees’ decisions to continue their careers or to leave their organizations [12]. Job stress reduces job satisfaction in hospitals, impeding the effective and efficient performance of healthcare workers and thereby directly impacting the quality of medical services [13]. Negative affectivity experienced by individuals in the workplace leads them to perceive their jobs as more stressful, and such stressors have been associated with turnover intention [14]. The literature contains numerous studies that demonstrate the relationship between job stress and turnover intention [10,14,15,16,17,18,19,20,21,22].

Regardless of whether turnover intention is solely attributed to job-related factors, it should be considered as a potential means of escaping stress [23]. Indeed, turnover intention represents a multifaceted psychological reaction to unfavorable work-related conditions, often expressed through withdrawal behaviors like planning to quit or actively seeking alternative employment [24].

Developing and bringing healthcare workers to a competent level is challenging and requires many years of effort. Therefore, in order to reduce healthcare employees’ turnover intentions, necessary measures must be taken to prevent workplace bullying, which contributes to increased pressure and, consequently, heightened job stress [25]. Workplace bullying indirectly intensifies stress and raises turnover rates among healthcare workers. This situation becomes a barrier to organizational progress and further threatens patient safety by diminishing the quality of healthcare services. Consequently, greater awareness and the development of solutions aimed at preventing workplace bullying are urgently needed [26]. Considering all these factors, healthcare institutions must reduce unnecessary workloads, develop certification systems that mitigate organizational stressors, and promote practices that foster employees’ positive commitment to the organization in order to prevent the departure of existing personnel [27].

In this study, research articles addressing the relationship between job stress and turnover intention in healthcare institutions were systematically reviewed and analyzed through meta-analytic techniques. Job stress has become a focal issue, particularly in professions such as nursing and medicine, which are recognized among the most demanding occupations. Healthcare organizations also report some of the highest turnover rates, highlighting the relevance of this topic.

The primary aim was to examine the association between job stress and turnover intention among healthcare employees. A systematic review and meta-analysis were conducted, targeting healthcare staff as the study population, job stress as the exposure, and turnover intention as the outcome variable. By synthesizing existing findings, this study highlights variations across prior research and provides a consolidated perspective that can inform future investigations. The research question guiding this analysis was “What is the overall association between job stress and turnover intention in healthcare organizations?

## 3. Methodology

### 3.1. Research Model

This study adopted a meta-analytical approach to statistically measure the association of job stress on turnover intention in healthcare organizations. This study protocol has been registered in the PROSPERO database (ID: CRD42024581906) to avoid duplication, facilitate comparisons with ongoing studies at the planning stage, and reduce bias. In the analysis of the studies included in this research, PRISMA (Preferred Reporting Items for Systematic Reviews and Meta-Analyses) guidelines were utilized as a framework to ensure critical appraisal and quality control in the integration of evidence-based research findings [28].

The present study followed the updated PRISMA 2020 guidelines [29], which. PRISMA 2020 provides a comprehensive framework for reporting the study selection process using a flow diagram, clearly documenting inclusion and exclusion criteria, and improving methodological transparency [30].

**Figure 1:**
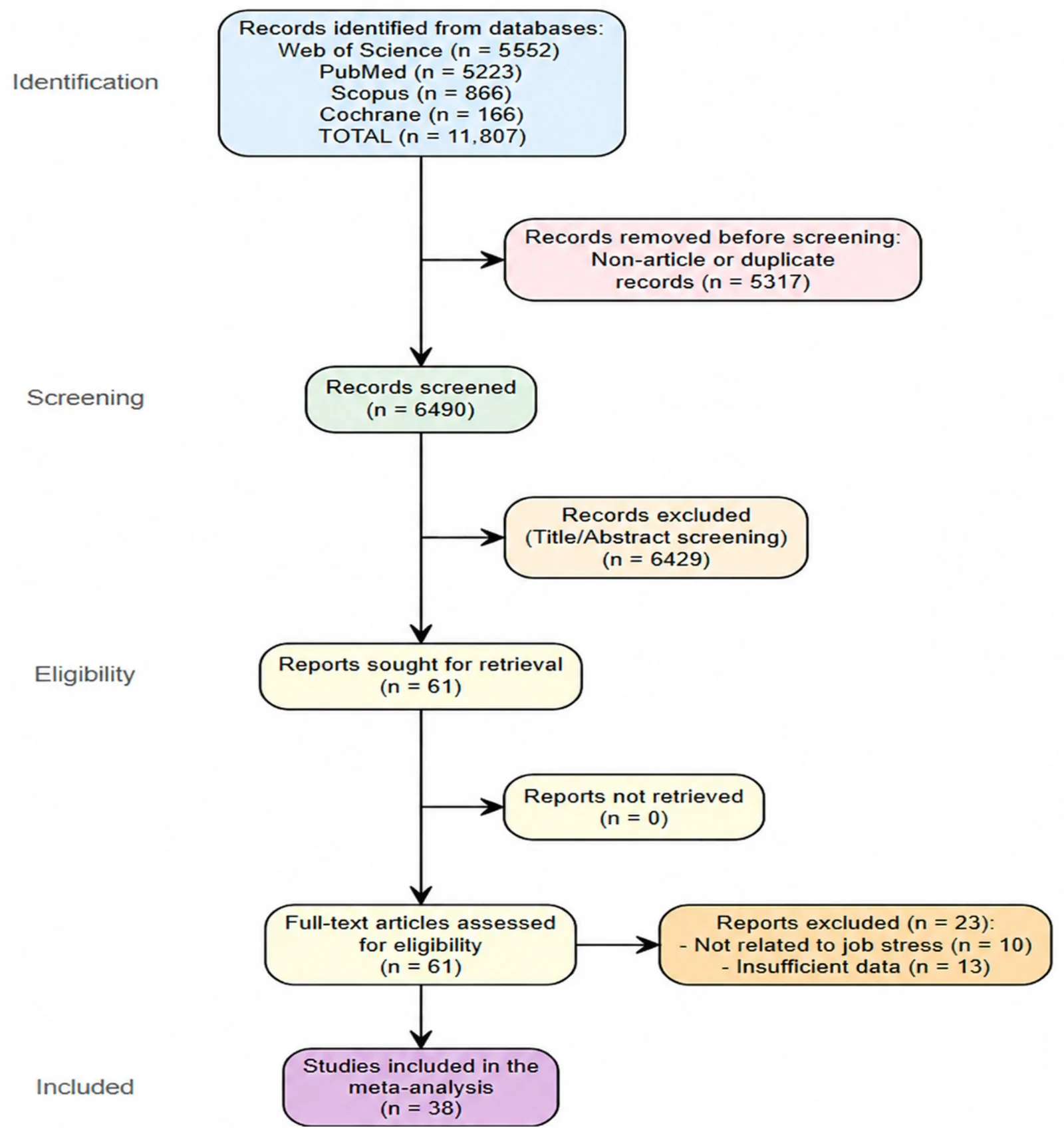
Flow diagram of the studies included in the analysis.

Given the sample sizes, publication years, and the variety of scales used, a random effects model was preferred in the analyses. The findings are first described using the flowchart (see Figure 1) and PRISMA 2020 flowchart (see Table A1), which outlines the study selection process. Then, a forest plot showing the general relationship between job stress and intention to leave, and publication bias analyses are presented. Subgroup analyses were conducted by occupational group, COVID-19 period, and geographic region to identify the sources of high heterogeneity among the studies. Meta-regression analyses were used to test the moderating roles of publication year on effect size.

### 3.2. Search Strategy

In this study, a literature search was conducted between 30 November 2025 and 20 December 2025 using the Scopus, WoS, PubMed and Cochrane databases. The search strategy targeted publications on job stress and turnover intention, using the following keyword combination: (“Job stress” OR “Work stress” OR “Occupational stress” OR “Workplace stress” OR “Job-related stress” OR “Work-related stress”) AND (“Turnover intention” OR “Intention to quit”).

A total of 11,807 studies related to these two topics were identified in the Web of Science, Scopus, PubMed, and Cochrane databases, of which 6490 were classified as articles.

### 3.3. Inclusion Criteria

The eligibility criteria were defined based on independent variables expected to influence the meta-analytic results and to quantify their effect [31,32]. Studies were included if they examined both job stress and turnover intention within healthcare institutions and employed quantitative, observational, survey-based designs. Only full-text journal articles published in Turkish or English were considered.

Inclusion criteria:

- Quantitative, survey-based studies available in full text in Turkish or English;
- Articles indexed in Scopus, Web of Science (WoS), PubMed, or Cochrane;
- Studies reporting sufficient statistical data to compute a common effect size (e.g., Pearson’s r or convertible statistics such as *t*, *F*, or regression coefficients);
- Research explicitly addressing the relationship between job stress and turnover intention in healthcare institutions.

Qualitative studies, theoretical papers, reviews and meta-analyses, case reports, and experimental or intervention-based designs (e.g., pre-test–post-test studies) were excluded. Studies that did not provide adequate statistical information for calculating or converting effect sizes were also excluded.

To ensure methodological consistency, all effect sizes were synthesized using Pearson’s correlation coefficient (r) as the common metric. Effect sizes derived from regression-based analyses were converted to r before meta-analytic synthesis in accordance with established meta-analytic procedures [28,33,34,35]. Studies for which effect sizes could not be reliably converted were excluded from the analysis. Duplicate records retrieved from multiple databases were identified, and only one instance of each study was retained. Following this process, 61 articles meeting the eligibility criteria were reviewed in detail. Data were systematically coded using Microsoft Excel, and 38 studies providing sufficient data were ultimately included in the Comprehensive Meta-Analysis (CMA), Version 3 & R Version 2026.05.1+225 softwares.

In the first stage of the meta-analysis process, a total of 11,807 records were identified as a result of comprehensive searches conducted through the WoS, PubMed, Scopus, and Cochrane databases. In the first stage of the meta-analysis process, a total of records were identified as a result of comprehensive searches conducted through the WoS, PubMed, Scopus, and Cochrane databases. From this large data pool, non-article-type publications and book chapters were excluded. Duplicate records were identified using Microsoft Excel sorting and filtering procedures and subsequently verified manually by comparing article titles, author names, publication years, and source information to ensure accurate duplicate removal. 6490 studies that advanced to the title and abstract search stage were examined in accordance with the purpose and scope of the research. Studies that did not directly address the relationship between job stress and intention to leave, or that were designed using qualitative methods, were eliminated at this stage. 61 studies deemed suitable for full-text review were subjected to a detailed evaluation. As a result of this evaluation, 23 studies with missing statistical data (sample size, r-value, or t/F values that could be converted) or focusing on irrelevant variables were excluded from the analysis. The remaining 38 studies that fully met the inclusion criteria were included in the quantitative synthesis stage. Every step of the selection process was conducted transparently to ensure the reproducibility and reliability of the study (Figure 1).

### 3.4. Data Coding

The processes of study screening, eligibility assessment, and data extraction were conducted independently by two reviewers. Discrepancies between reviewers were resolved through discussion and consensus. When consensus could not be reached, a third reviewer was consulted to make the final decision. This procedure was applied to ensure the accuracy and reliability of the screening and coding processes.

After identifying the quantitative studies related to job stress and turnover intention in healthcare institutions that met the inclusion criteria, the studies were systematically and explicitly coded to ensure compatibility with meta-analytic procedures. Microsoft Excel was used as the primary tool for data coding and organization.

A standardized coding form was developed in Excel and consisted of three main sections. The first section captured bibliographic information related to the identity of the studies (e.g., author(s), publication year). The second section included descriptive information regarding study characteristics, such as sample size and professional group. The third section contained the statistical information required for meta-analysis, including effect size–related data reported in the original studies. All coded data were subsequently transferred to the Comprehensive Meta-Analysis (CMA) & R softwares for statistical synthesis.

## 4. Results

### 4.1. Data Analysis

Given the substantial heterogeneity among the included studies (Q = 819.85, *p* < 0.001; I^2^ = 95.5%), a random-effects model was applied in all analyses. Information regarding the studies examining the association between job stress and turnover intention in healthcare institutions is presented in Table 1.

As shown in Table 1, a total of 38 studies related to the topic were included in the meta-analysis. The included studies were published between 2009 and 2025, with a relatively balanced distribution across years. Among the 38 studies, samples consisted of nurses (*n* = 21), mixed healthcare personnel (*n* = 9), physicians (*n* = 5), and therapists (*n* = 3). Twelve of these studies were conducted in the Middle East region, 22 in East Asia, and 4 in the West/Other region. In this study, the articles included were not selected based on their country of origin; all articles found in relevant databases and meeting the necessary inclusion criteria were included.

### 4.2. Evaluating Quality of the Studies

To assess the quality and risk of bias in cross-sectional studies, the AXIS evaluation tool was used. The AXIS evaluation tool consists of 20 questions, with a total score ranging from 0 to 20. An increase in the total score indicates improved study quality [73]. Furthermore, the risk of bias was reviewed by two independent researchers and assessed at the outcome level, as in the study by Sayık et al. [74]. Again, disagreements between reviewers were resolved through discussion or with the participation of a third reviewer. Table 1 shows that all 38 studies included in the research generally had high quality scores.

### 4.3. Ensuring the Reliability and Validity of the Research

To ensure the reliability of the research, coding was conducted independently by two different individuals, and reliability was subsequently calculated. For the reliability calculation, the formula Reliability = [Agreement/(Agreement + Disagreement)] × 100 was used. The calculated value, 91%, indicates that the level of reliability was sufficient [75].

In line with the inclusiveness principle of meta-analysis, all these studies that met the inclusion criteria were incorporated into the research to prevent publication bias. As a result of the searches conducted in the aforementioned databases, 38 articles that satisfied the specified inclusion criteria were included in the analysis. Publication bias is seen as a threat to the external validity of meta-analyses [76].

**Figure 2:**
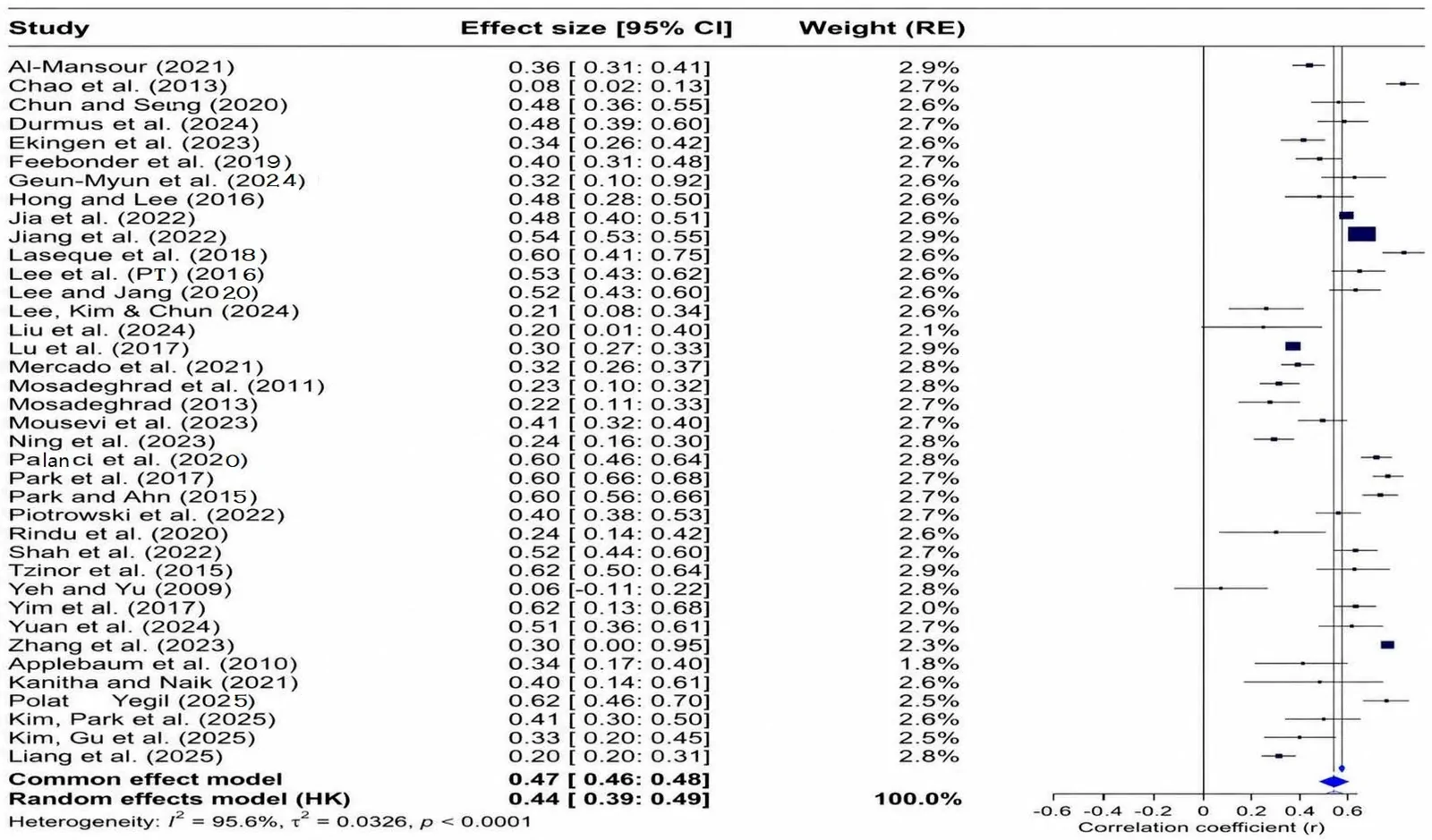
Forest plot of the relationship between job stress and intention to leave (general model). Studies included: [36,37,38,39,40,41,42,43,44,45,46,47,48,49,50,51,52,53,54,55,56,57,58,59,60,61,62,63,64,65,66,67,68,69,70,71,72]. Note: Since Kim et al. [70] and Kim et al. [71] are the author of two different studies by two different authors, the terms Kim, Park et al. (2025) [71] and Kim, Gu et al. (2025) [70] are used to understand the difference between the studies.

The overall effect size obtained by synthesizing independent studies examining the relationship between job stress experienced by healthcare workers and their intention to leave their jobs demonstrates a positive, moderate-to-high correlation between these two variables [(r = 0.441, 95% CI [0.389, 0.490], *p* < 0.0001)] (Figure 2). Examining the confidence intervals of each study on the forest plot reveals that the direction of the effect is consistent across all studies, with the intention to leave the job inevitably increasing as stress levels rise. This coefficient, calculated using a random-effects model, indicates that job stress is an important psychosocial factor associated with turnover intention among healthcare workers. The intense workload, emotional pressure, and time constraints inherent in healthcare services exhaust employees’ coping mechanisms, leading them to consider career changes.

The high variation observed among the analyzed studies [(I^2^ = 95.5%)] indicates that the strength of the relationship between job stress and intention to leave varies depending on contextual factors. The Q statistic obtained from heterogeneity tests [(Q = 819.85, *p* < 0.0001)] shows that the difference between studies is too large to be explained by chance and necessitates the presence of moderator variables. Despite the use of different methodologies and scales, the overall effect is statistically significant, and the confidence interval is narrow.

The observed association reflects stressors operating at two interrelated levels within healthcare settings. At the institutional level, job stress is shaped by organizational structures, management practices, workload distribution, and human resource policies within healthcare institutions. At the service level, stress also arises from the nature of healthcare service delivery itself, including patient care intensity, emotional labor, time pressure, and continuous exposure to clinical demands. Therefore, the pooled effect size captures the combined influence of both institutional and service-related stressors inherent in healthcare work.

**Figure 3:**
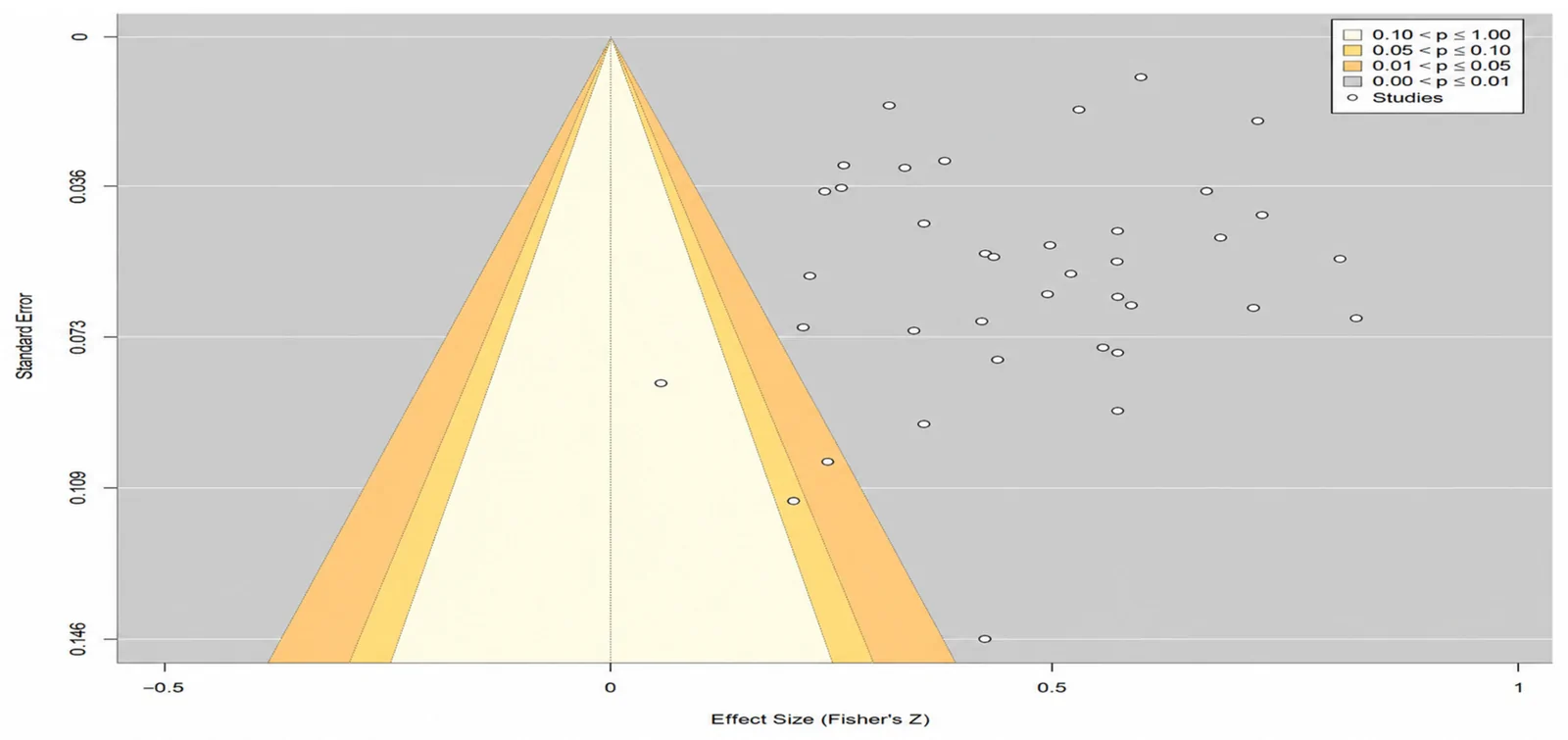
Funnel plot for evaluating publication bias.

In this study, the “funnel plot” was initially used to assess the potential for publication bias and visual inspection of the funnel plot. The results of the funnel plot are presented in Figure 3. In a funnel plot, the *y*-axis represents the standard errors of the studies, while the *x*-axis shows the effect sizes. Studies with low standard errors are concentrated near the average effect size and at the top of the plot. In contrast, studies with high standard errors (lower precision) are distributed toward the bottom of the plot. In meta-analysis, a symmetric distribution of studies on both sides of the vertical reference line representing the combined effect size is considered an indicator of the absence of publication bias [77,78].

Visual inspection of the funnel plot suggests some degree of asymmetry in the distribution of effect sizes (Figure 3). However, given the substantial between-study heterogeneity (I^2^ = 95.5%), this apparent asymmetry should be interpreted with caution, as heterogeneity itself may contribute to the observed pattern. Therefore, publication bias cannot be conclusively inferred from visual inspection alone.

The fact that studies with small sample sizes are spread across a wide area at the bottom of the funnel plot suggests the presence of a small study effect.

**Figure 4:**
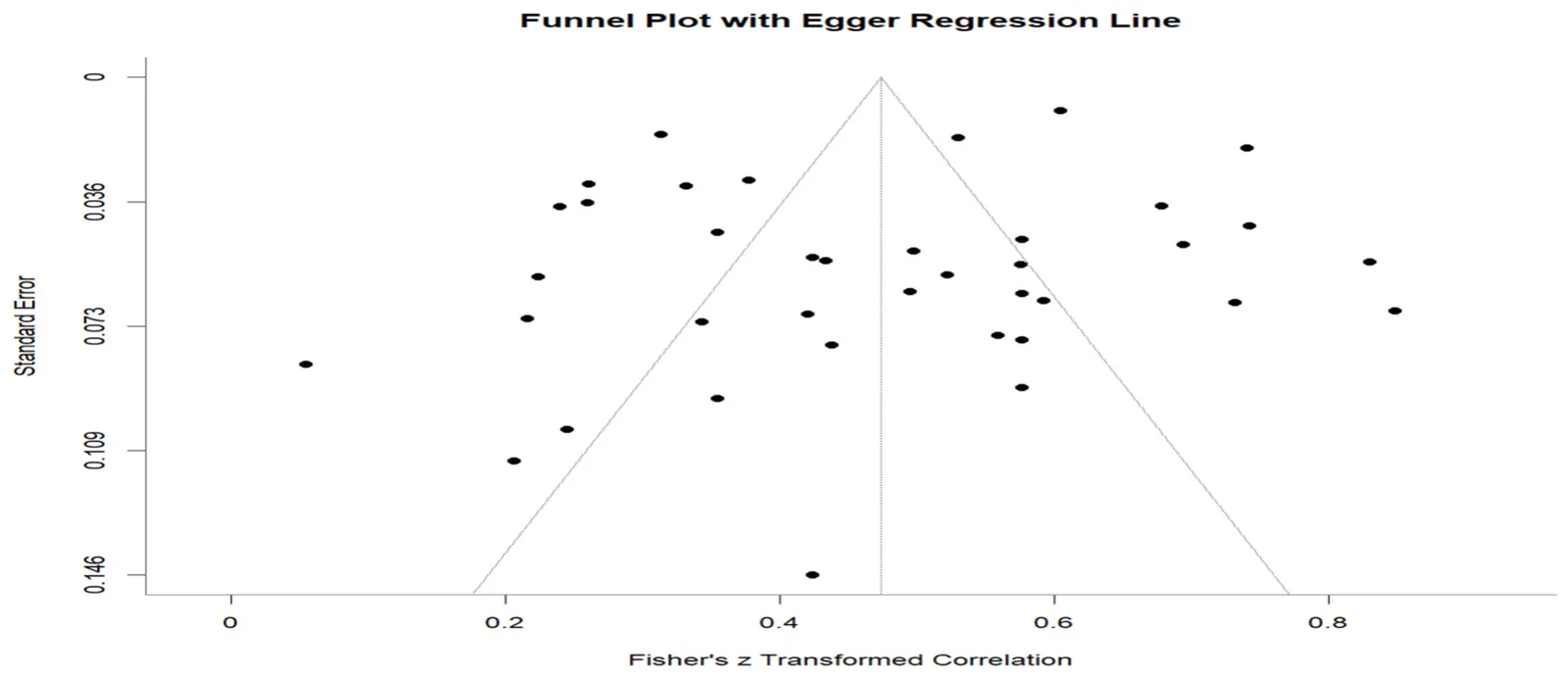
Funnel plot with Egger’s Regression Line.

In addition to funnel plot analysis, Egger’s regression test was also performed in the study (Figure 4). Accordingly, the symmetry of the distribution and the insignificance of the regression test results (*p* = 0.3045) indicate that there is no publication bias.

**Figure 5:**
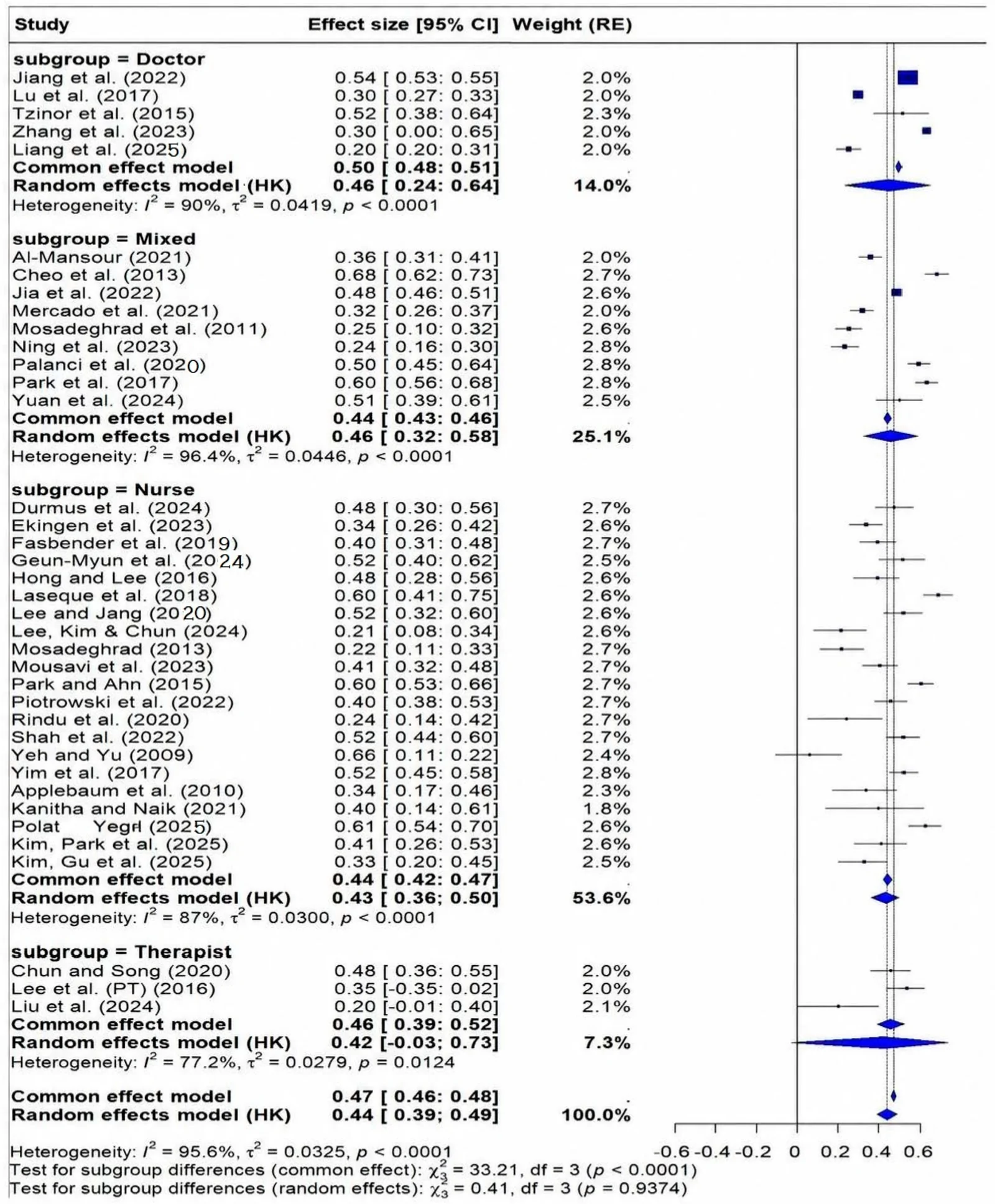
Forest plot: subgroup analysis by occupational groups. Studies included: [36,37,38,39,40,41,42,43,44,45,46,47,48,49,50,51,52,53,54,55,56,57,58,59,60,61,62,63,64,65,66,67,68,69,70,71,72].

Subgroup analyses conducted specifically for occupational groups showed that the association between job stress and turnover intention was stronger among physicians compared to other healthcare professionals (r = 0.46) (Figure 5). Although physicians exhibited a numerically higher effect size than some other occupational groups, the between-group difference was not statistically significant (*p* = 0.94). Therefore, this finding should be interpreted with caution. While previous studies have suggested that factors such as decision-making responsibilities, long working hours, and professional mobility may contribute to work-related stress among physicians, the present meta-analysis does not provide sufficient evidence to conclude that the association between job stress and turnover intention differs significantly across occupational groups.

Although the effect size calculated for nurses is lower than that of physicians, it is statistically quite strong [(r = 0.43)]. Nurses, who are at the center of patient care and bear the greatest emotional labor burden, are able to use professional solidarity and social support mechanisms more actively in coping with stress. A similar trend was observed in the mixed group including other healthcare professionals, and the difference between the professional groups was found to be statistically insignificant [(*p* = 0.94)].

**Figure 6:**
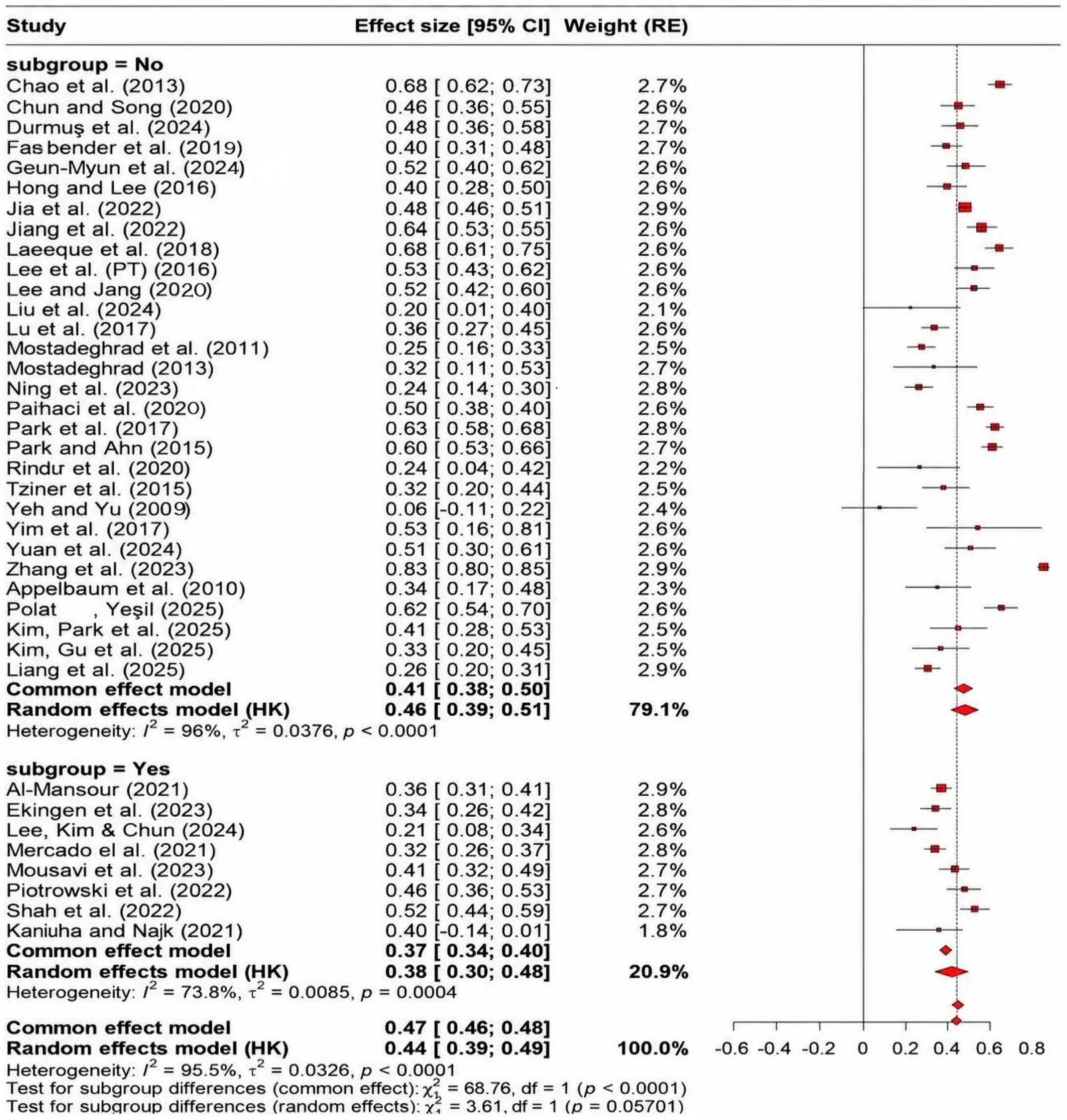
Forest plot of subgroup analysis before and during the COVID-19 period. Studies included: [36,37,38,39,40,41,42,43,44,45,46,47,48,49,50,51,52,53,54,55,56,57,58,59,60,61,62,63,64,65,66,67,68,69,70,71,72].

A period analysis examining the impact of the pandemic on work dynamics surprisingly revealed that pre-pandemic studies exhibited a higher correlation coefficient compared to the pandemic period [(r = 0.46 and r = 0.38, respectively)] (Figure 6). Although the effect sizes appeared to differ between studies conducted during and outside the COVID-19 period, the between-group difference was not statistically significant (*p* = 0.05). Therefore, these findings should be interpreted with caution. The previous literature has suggested that factors such as economic uncertainty, job security concerns, and limited alternative employment opportunities during periods of crisis may influence employees’ intentions to remain in their current positions despite stressful working conditions. However, the present meta-analysis does not provide sufficient evidence to conclude that the association between job stress and turnover intention differed significantly according to the COVID-19 period.

The intense sense of moral responsibility and social solidarity felt by healthcare workers during this period was another factor limiting their intention to leave. Employees on the front lines of the pandemic perceived continuing their duties under stressful conditions as a necessity due to their professional ethical values. In the pre-pandemic period, the more flexible labor market and abundance of alternatives allowed employees to show less tolerance for stress factors and make the decision to leave more quickly. The fact that the statistical difference between the groups remained at a marginal level [(*p* = 0.05)] proves that crisis periods do not completely sever the stress–separation relationship, but modify the dynamics of the relationship according to the periodic conditions.

**Figure 7:**
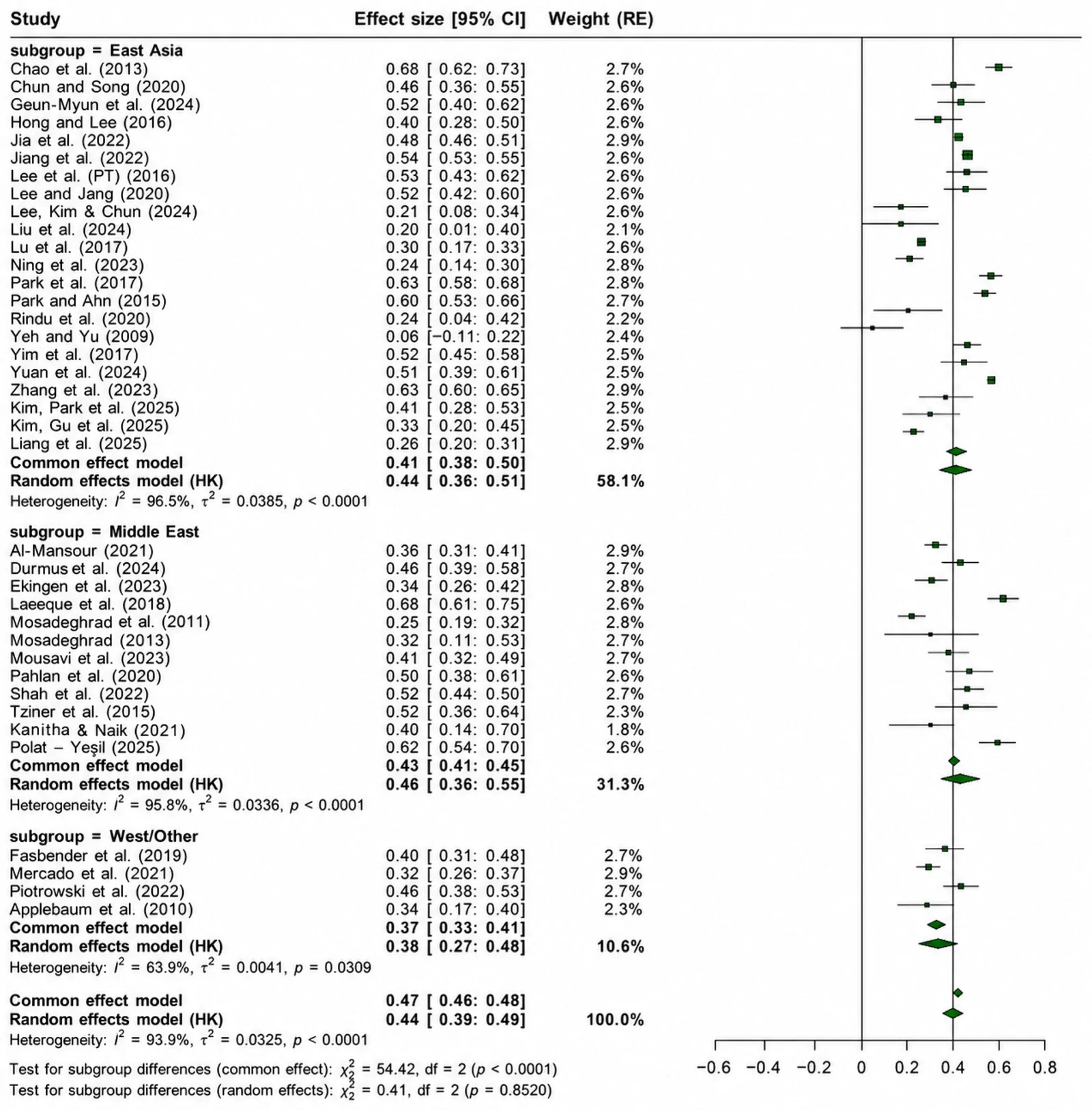
Subgroup analysis of forests plot by geographic regions. Studies included: [36,37,38,39,40,41,42,43,44,45,46,47,48,49,50,51,52,53,54,55,56,57,58,59,60,61,62,63,64,65,66,67,68,69,70,71,72].

A geographical analysis revealed that studies originating from East Asia and the Middle East showed a marginally higher correlation with studies originating from the West [(East Asia r = 0.44, Middle East r = 0.46, West/Other r = 0.38)] (Figure 7). Although some variation in effect sizes was observed across geographical regions, the between-group difference was not statistically significant (*p* = 0.28). Therefore, these findings should be interpreted with caution. Previous studies have suggested that cultural characteristics, organizational norms, and workplace structures may influence employees’ experiences of work-related stress and turnover intention. However, the present meta-analysis does not provide sufficient evidence to conclude that the association between job stress and turnover intention differs significantly across geographical regions.

The lack of a statistically significant difference between groups [(*p* = 0.28)] documents that intention to leave the job due to work stress is a global workforce problem that transcends cultural boundaries. The universal nature of healthcare delivery and the inherent challenges of dealing with human health lead employees to exhibit similar responses regardless of their geographical location. In developing countries, resource scarcity and infrastructure problems are the main factors fuelling stress, while in developed countries, bureaucratic burdens and legal responsibilities take center stage.

**Figure 8:**
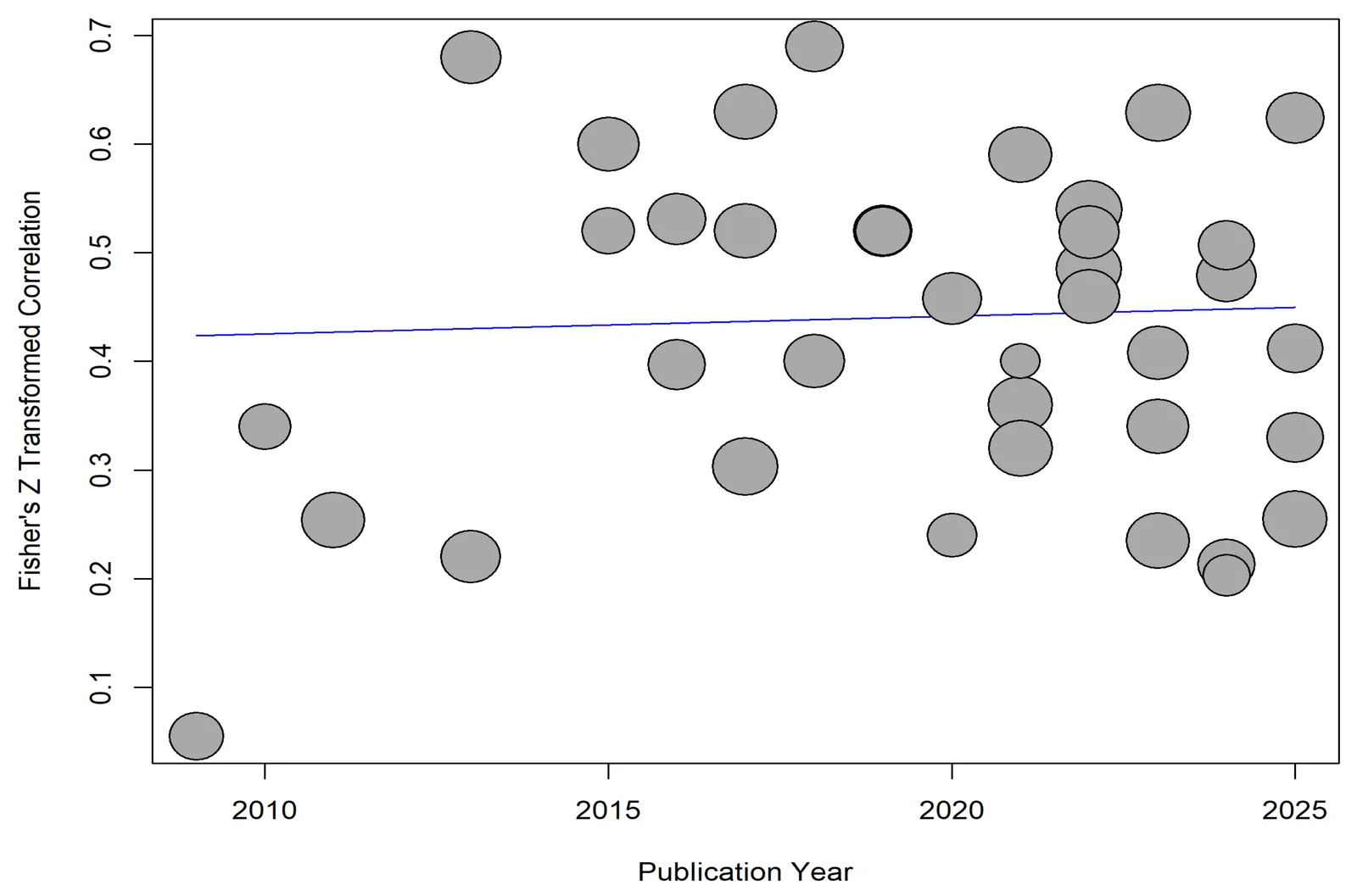
Meta-regression (bubble) graph showing the association of the year of publication on the relationship between job stress and intention to leave.

A meta-regression analysis examining whether publication year moderated the relationship between job stress and turnover intention showed that publication year was not a significant moderator of this relationship (QM = 0.0779, *p* = 0.78). Changing the years in which the studies were conducted did not statistically significantly alter the intensity of stress-related resignation decisions among healthcare workers. Technological advancements or managerial reforms in healthcare systems over time have been insufficient to mitigate the devastating effects of stress on resignation. The chronicity of structural problems in the healthcare sector throughout the examined time period caused the relationship to remain stable over the years. Even periodic fluctuations caused by global crises such as pandemics were not enough to break the strength of this fundamental link between stress and intention to leave (Figure 8).

## 5. Discussion

This meta-analysis aimed to examine the relationship between job stress and intention to leave in healthcare institutions through a synthesis of quantitative research and to contribute to the field of healthcare management based on the findings. A total of 38 articles obtained from relevant databases were comprehensively analyzed in this study.

Before presenting the practical and policy recommendations, it is important to interpret the findings of this study within a quantitative meta-analytic framework. The pooled effect size obtained in this meta-analysis (r = 0.441; 95% CI [0.389, 0.490]) demonstrates that job stress constitutes a moderate-to-strong and statistically robust psychosocial risk factor for turnover intention in healthcare settings, rather than a context-specific or marginal influence. Moreover, although between-study heterogeneity was substantial (I^2^ = 95.5%), the direction and statistical significance of the association remained consistent across different countries, healthcare systems.

From an interpretative perspective, the findings of this meta-analysis highlight the dual nature of job stress in healthcare settings. While the analysis is framed at the level of healthcare institutions, the observed relationship between job stress and intention to leave also reflects stressors embedded in the healthcare service delivery process. Organizational factors such as management practices, staffing policies, and institutional workload structures interact with service-related demands, including patient care intensity, emotional labor, and time pressure.

The findings of the present meta-analysis are consistent with established theoretical models of occupational stress, particularly the Job Demand-Control Model [79] and the Effort–Reward Imbalance Model [80]. These frameworks suggest that excessive job demands, limited control over work processes, and inadequate rewards increase psychological strain and may contribute to withdrawal-related outcomes. The moderate-to-strong positive association observed in this study supports the argument that job stress is an important antecedent of turnover intention among healthcare workers.

The findings are also consistent with previous empirical studies conducted among healthcare professionals. Several studies included in this meta-analysis reported that workload, emotional demands, role conflict, insufficient organizational support, and work-related pressure were associated with higher turnover intentions. The present meta-analysis extends this evidence by demonstrating that the relationship remains statistically significant across different healthcare systems, occupational groups, and geographical regions. This consistency suggests that job stress represents a common organizational risk factor influencing workforce retention in healthcare settings.

The findings suggest that the relationship between job stress and turnover intention is not confined to a specific country, healthcare system, or occupational group. The consistency of the observed association across diverse study contexts indicates that job stress may represent a widespread workforce challenge in healthcare settings. Previous studies have similarly reported that prolonged exposure to occupational stressors is associated with burnout, reduced organizational commitment, and stronger intentions to leave among healthcare professionals.

Organizational environments characterized by excessive workload, insufficient support, and limited attention to employee well-being may contribute to chronic stress and higher turnover intentions among healthcare workers. Similarly, difficulties in balancing workloads and providing fair working conditions may weaken employees’ organizational commitment and sense of belonging. These factors may ultimately increase workforce instability and create challenges for healthcare organizations seeking to retain experienced personnel.

The intention to leave a job, when acted upon, represents a high cost and quality loss for healthcare organizations, beyond just personnel loss. Attempting to fill the void created by the departure of experienced personnel with recent graduates leads to a decrease in service quality and an increase in the risk of medical errors. The extra workload placed on remaining employees exacerbates existing stress, creating a vicious cycle. Given the labor-intensive nature of healthcare, protecting human resources is essential for maintaining both organizational sustainability and service quality.

Beyond individual interventions aimed at increasing employees’ psychological resilience, fundamental organizational changes are needed. Adopting flexible working models and strengthening social support mechanisms play a vital role in buffering the destructive effects of stress. Participatory management models, where employees feel valued and can participate in decision-making processes, are among the approaches that can potentially contribute to reducing turnover intentions. Strengthening internal communication and increasing professional development opportunities help employees regain their motivation.

Prioritizing strategies for protecting human resources in the process of formulating health policies is essential for preparing for future health crises. Nationally planned health reforms need to be expanded to include employee well-being in addition to infrastructure and technology investments. It is considered that the introduction of legal regulations and standards to combat work stress can offer a protective framework for healthcare workers against burnout syndrome. It is a critical turning point for the long-term success of the system that policymakers view the healthcare workforce not as a resource, but as a strategic asset to be protected. A sustainable healthcare system can only be built on physically and mentally healthy employees.

Although the pooled correlation indicates a moderate positive association between job stress and turnover intention, the certainty of this estimate should be interpreted with caution. The substantial between-study heterogeneity (I^2^ = 95.5%) suggests considerable variability in study populations, healthcare settings, occupational groups, and measurement instruments. Furthermore, the evidence is derived exclusively from cross-sectional studies, which preclude causal inference. Therefore, while the overall association appears robust across studies, the precise magnitude of the pooled effect should be interpreted with moderate caution, and future high-quality longitudinal studies are needed to strengthen the certainty of the evidence.

## 6. Conclusions

This systematic review and meta-analysis demonstrated a statistically significant positive association between job stress and turnover intention among healthcare workers. Higher levels of job stress were associated with a greater likelihood of intending to leave the organization. Although substantial heterogeneity was observed across studies, the relationship remained consistent across occupational groups, regions, and study contexts, indicating that this association is robust across diverse healthcare settings.

These findings suggest that job stress is not merely an individual psychological concern but also an important organizational challenge with implications for workforce sustainability. Given the labor-intensive nature of healthcare services, the retention of qualified healthcare professionals is essential for maintaining service quality, organizational effectiveness, and continuity of care. Persistent job stress may increase the risk of workforce instability by strengthening employees’ intentions to leave their organizations.

From a healthcare management perspective, the findings emphasize the importance of developing organizational strategies aimed at reducing work-related stress and promoting employee well-being. Efforts to improve working conditions, provide supportive management practices, and strengthen psychosocial support mechanisms may contribute to retaining healthcare professionals and supporting the long-term sustainability of healthcare systems.

## 7. Limitations

Several limitations of this study should be acknowledged. Although extensive subgroup and meta-regression analyses were conducted to explore potential sources of heterogeneity, a high level of between-study heterogeneity remained (I^2^ = 95.5%). This suggests that additional unmeasured contextual or methodological factors—such as institutional characteristics, health system structures, workload intensity, or variations in measurement instruments—may have influenced the observed effect sizes and could not be fully captured in the present analyses.

Second, all included studies employed cross-sectional and observational survey designs. Therefore, the findings of this meta-analysis should be interpreted as associational rather than causal in nature. Although a robust and statistically significant relationship between job stress and turnover intention was identified, the present study does not permit causal inferences regarding the directionality of this relationship or the effectiveness of specific organizational interventions.

Accordingly, the practical and policy implications discussed in this study should be interpreted as evidence-informed and risk-oriented considerations, rather than empirically tested or prescriptive solutions. Future research employing longitudinal, experimental, or intervention-based designs, as well as more refined moderator and mediation analyses, is required to clarify causal mechanisms and to evaluate the effectiveness of targeted stress-reduction strategies in healthcare settings.

In addition to the limitations noted above, several further issues should be considered when interpreting the findings of this meta-analysis. First, a substantial proportion of the included studies were conducted in Asian countries, which may introduce cultural bias and limit the generalizability of the findings to healthcare systems operating in different cultural and organizational contexts. Second, the inclusion of studies published across a wide time span may have introduced temporal bias, as changes in healthcare systems, working conditions, and external shocks such as the COVID-19 pandemic could have influenced the strength of the observed association over time. Finally, the use of different measurement instruments to assess job stress and turnover intention across studies may have resulted in instrument bias, as variations in scale content and psychometric properties could have affected the comparability of effect sizes.

A further limitation is that all included studies relied on self-report questionnaires administered at a single time point to measure both job stress and turnover intention. Such a design may introduce common-method bias and inflate the observed association between these variables. Therefore, the pooled effect size should be interpreted with caution.

A further limitation of this study is that the search strategy did not explicitly include all possible synonymous terms for turnover intention (e.g., intention to leave or intent to leave). Although the selected search terms identified studies relevant to the review question, the omission of additional synonymous terms may have resulted in the exclusion of some potentially eligible studies.

Another limitation is that inter-rater agreement was assessed using percentage agreement rather than a chance-corrected statistic such as Cohen’s kappa. Therefore, the reported agreement may overestimate the true level of agreement between reviewers.

## 8. Recommendations

In this context, the findings support recommendations at three distinct levels:

### 8.1. Practitioners and Human Resources Professionals

- Stress management training should become an integral part of organizational culture, incorporating psychological counseling, individual support sessions, and mindfulness-based practices.
- In addition to organizational stress management strategies, mindfulness-based interventions (MBIs) are among the approaches supported by independent empirical evidence for reducing stress in healthcare settings. A recent systematic review and meta-analysis encompassing 27 studies and more than 2500 healthcare professionals reported that mindfulness-based interventions produced significant short-term reductions in stress, anxiety, burnout, and psychological distress, with small-to-moderate effect sizes across multiple well-being outcomes [81]. In this context, considering the quantitatively robust association between job stress and turnover intention identified in the present meta-analysis, mindfulness-based practices may be regarded as complementary approaches that could indirectly contribute to managing turnover risk through stress reduction, rather than as direct or causal interventions.
- Shift systems and workloads should be reorganized to implement fairer scheduling models.
- Systems such as employee satisfaction surveys and exit interviews should be used to detect turnover intention in advance.
- Psychosocial support programs aimed at enhancing emotional resilience should be developed.

### 8.2. Healthcare Institution Managers and Policymakers

- Employee engagement should be enhanced through reward and recognition systems; corporate loyalty programs and work–life balance initiatives should be implemented.
- The Ministry of Health and relevant institutions should establish regulations to combat job stress (e.g., limits on shift durations, rest rights).
- Institutional systems to monitor stress levels should be developed in public hospitals, and these data should be used in managerial decision-making.

### 8.3. Academics and Researchers

- Future studies should expand the model by including moderator and mediator variables (such as organizational support, leadership style, job satisfaction, etc.).
- Subgroup analyses should be conducted based on different professional groups, with distinctions made for nurses, doctors, administrative staff, etc.
- Findings should be deepened through mixed-methods studies that combine qualitative and quantitative approaches.
- International comparisons should be made to evaluate the impact of cultural differences, and comparisons of different organizational structures should be conducted.

Overall, the findings of this systematic review and meta-analysis indicate that job stress is a significant factor associated with turnover intention among healthcare workers and highlight the importance of organizational strategies aimed at reducing work-related stress and supporting workforce retention.

## Figures and Tables

**Table 1:** Characteristics of the studies.

Study	COR *	N **	Profession	COVID ***	Region	AXIS Score (Max 20)	Quality Level	Design
Al-Mansour (2021) [36]	0.36	1101	Mixed	Yes	Middle East	18	High	Cross-sectional
Chao et al. (2013) [37]	0.68	344	Mixed	No	East Asia	16	High	Cross-sectional
Chun and Song (2020) [38]	0.458	257	Therapist	No	East Asia	17	High	Cross-sectional
Durmuş et al. (2024) [39]	0.479	302	Nurse	No	Middle East	18	High	Cross-sectional
Ekingen et al. (2023) [40]	0.34	486	Nurse	Yes	Middle East	17	High	Cross-sectional
Fasbender et al. (2019) [23]	0.4	361	Nurse	No	West/Other	18	High	Cross-sectional
Geun-Myun et al. (2024) [41]	0.52	172	Nurse	No	East Asia	16	High	Cross-sectional
Hong and Lee (2016) [42]	0.397	211	Nurse	No	East Asia	15	High	Cross-sectional
Jia et al. (2022) [43]	0.485	3191	Mixed	No	East Asia	18	High	Cross-sectional
Jiang et al. (2022) [44]	0.54	10,457	Doctor	No	East Asia	19	High	Cross-sectional
Laeeque et al. (2018) [45]	0.69	216	Nurse	No	Middle East	16	High	Cross-sectional
Lee et al. (PT) (2016) [46]	0.531	236	Therapist	No	East Asia	15	High	Cross-sectional
Lee and Jang (2020) [47]	0.52	252	Nurse	No	East Asia	17	High	Cross-sectional
Lee, Kim and Chun (2024) [48]	0.213	203	Nurse	Yes	East Asia	18	High	Cross-sectional
Liu et al. (2024) [49]	0.203	82	Therapist	No	East Asia	15	High	Cross-sectional
Lu et al. (2017) [50]	0.303	3563	Doctor	No	East Asia	18	High	Cross-sectional
Mercado et al. (2021) [51]	0.32	985	Mixed	Yes	West/Other	17	High	Cross-sectional
Mosadeghrad et al. (2011) [52]	0.254	740	Mixed	No	Middle East	16	High	Cross-sectional
Mosadeghrad (2013) [53]	0.22	296	Nurse	No	Middle East	15	High	Cross-sectional
Mousavi et al. (2023) [54]	0.408	350	Nurse	Yes	Middle East	17	High	Cross-sectional
Ning et al. (2023) [55]	0.235	703	Mixed	No	East Asia	18	High	Cross-sectional
Palancı et al. (2020) [56]	0.59	708	Mixed	No	Middle East	16	High	Cross-sectional
Park et al. (2017) [57]	0.63	532	Mixed	No	East Asia	17	High	Cross-sectional
Park and Ahn (2015) [58]	0.6	419	Nurse	No	East Asia	16	High	Cross-sectional
Piotrowski et al. (2022) [59]	0.46	390	Nurse	Yes	West/Other	17	High	Cross-sectional
Rindu et al. (2020) [60]	0.24	97	Nurse	No	East Asia	15	High	Cross-sectional
Shah et al. (2022) [61]	0.519	335	Nurse	Yes	Middle East	17	High	Cross-sectional
Tziner et al. (2015) [62]	0.52	124	Doctor	No	Middle East	16	High	Cross-sectional
Yeh and Yu (2009) [63]	0.055	144	Nurse	No	East Asia	15	High	Cross-sectional
Yim et al. (2017) [64]	0.52	447	Nurse	No	East Asia	17	High	Cross-sectional
Yuan et al. (2024) [65]	0.507	178	Mixed	No	East Asia	18	High	Cross-sectional
Zhang et al. (2023) [66]	0.629	2358	Doctor	No	East Asia	19	High	Cross-sectional
Applebaum et al. (2010) [67]	0.34	116	Nurse	No	West/Other	15	High	Cross-sectional
Kanitha and Naik (2021) [68]	0.4	50	Nurse	Yes	Middle East	14	Middle	Cross-sectional
Polat and Yesil (2025) [69]	0.624	232	Nurse	No	Middle East	19	High	Cross-sectional
Kim et al. (2025) [70]	0.412	165	Nurse	No	East Asia	18	High	Cross-sectional
Kim et al. (2025) [71]	0.33	198	Nurse	No	East Asia	18	High	Cross-sectional
Liang et al. (2025) [72]	0.255	1024	Doctor	No	East Asia	19	High	Cross-sectional

Note: Cor * = correlation coefficient, N ** = sample size, COVID *** = It provides information on whether the research is related to COVID-19 or not, Region = Geographical region of the study. These are different studies from the one below.

## Data Availability

No new data were created or analyzed in this study. Data sharing is not applicable to this article.

## Appendix A

**Table A1:** PRISMA 2020.

Section and Topic	Item	Checklist Item	Location Where Item Is Reported
**TITLE**			
Title	1	A systematic literature review and meta-analysis were conducted for this research. The title of this study is “The Association Between Job Stress and Intention to Leave in Healthcare Institutions: A Systematic Review and Meta-Analysis ”.	1
**ABSTRACT**			
Abstract	2	This research aimed to examine the association of job stress on turnover intention among employees working in healthcare institutions through a quantitative meta-analysis. In this study, a literature search was conducted between 30 November 2025, and 20 December 2025 using the Scopus, Web of Science, PubMed and Cochrane databases. Among the criteria for including studies in the literature on the subject in this research are the following: Studies that examine both job stress and intention to leave in healthcare settings have been included. Studies conducted on healthcare professionals (e.g., nurses, physicians, therapists, or a diverse sample of healthcare personnel) were included. Studies with quantitative, observational, and survey-based research designs were included. Full-text published, peer-reviewed journal articles were included in the study. Studies indexed in Scopus, Web of Science, PubMed, and Cochrane databases were included. Studies published in either English or Turkish have been included. Studies reporting sufficient statistical information to allow the calculation of a common effect size (e.g., Pearson correlation coefficient (r) or r-convertible statistics such as t, F, regression coefficients) were included. In this study, since all 38 articles included were cross-sectional studies, the AXIS assessment tool was used to evaluate study quality and risk of bias. Based on predefined inclusion criteria, 38 independent studies were included. Data were systematically coded using Microsoft Excel, and 38 studies providing sufficient data were ultimately included in the Comprehensive Meta-Analysis (CMA) & R softwares. Considering variations in sample sizes, publication years, and measurement scales, a random-effects model was employed. Effect sizes were illustrated with a forest plot, and publication bias analyses were performed. Findings revealed a moderate-to-high, positive, and statistically significant relationship (r = 0.441; 95% CI [0.389, 0.490]) between job stress and turnover intention among healthcare workers. Moreover, although between-study heterogeneity was substantial (I^2^ = 95.5%), the direction and statistical significance of the association remained consistent across different countries, healthcare systems, and professional groups. Several limitations of this study should be acknowledged. Although extensive subgroup and meta-regression analyses were conducted to explore potential sources of heterogeneity, a high level of between-study heterogeneity remained (I^2^ = 95.5%). This suggests that additional unmeasured contextual or methodological factors—such as institutional characteristics, health system structures, workload intensity, or variations in measurement instruments—may have influenced the observed effect sizes and could not be fully captured in the present analyses. First, a substantial pro-portion of the included studies were conducted in Asian countries, which may introduce cultural bias and limit the generalizability of the findings to healthcare systems operating in different cultural and organizational contexts. Second, the inclusion of studies published across a wide time span may have introduced temporal bias, as changes in healthcare systems, working conditions, and external shocks such as the COVID-19 pandemic could have influenced the strength of the observed association over time. Finally, the use of different measurement instruments to assess job stress and turnover intention across studies may have resulted in instrument bias, as variations in scale content and psychometric properties could have affected the comparability of effect sizes. Given the limited number of meta-analyses in healthcare management and human resources literature, this study provides a meaningful contribution by clarifying the relationships of job stress with turnover intention and highlighting the importance of addressing stress in healthcare institutions. This research received no external funding. The study was registered in the International Prospective Register of Systematic Reviews (PROSPERO; ID: CRD42024581906).	1, 6, 20, 9, 12, 17, 18
**INTRODUCTION**			
Rationale	3	In recent years, job stress and turnover intention have become focal points of attention. Professions in the healthcare sector, such as nursing and medicine, are among the most stressful fields, and healthcare institutions are reported to have some of the highest employee turnover rates. Therefore, in this study, articles focusing on job stress and turnover intention within healthcare organizations have been systematically examined.	1, 2
Objectives	4	This study aimed to examine the association of job stress with turnover intention among employees working in healthcare institutions through a quantitative meta-analysis.	1
**METHODS**			
Eligibility criteria	5	The inclusion criteria for this study are as follows: Studies that examine both job stress and intention to leave in healthcare settings have been included. Studies conducted on healthcare professionals (e.g., nurses, physicians, therapists, or a diverse sample of healthcare personnel) were included. Studies with quantitative, observational, and survey-based research designs were included. Full-text published, peer-reviewed journal articles were included in the study. Studies indexed in Scopus, Web of Science, PubMed, and Cochrane databases were included. Studies published in either English or Turkish have been included. Studies reporting sufficient statistical information to allow the calculation of a common effect size (e.g., Pearson correlation coefficient (r) or r-convertible statistics such as t, F, regression coefficients) were included. Exclusion criteria for this study are as follows: Qualitative research, theoretical studies, reviews, and meta-analyses. Experimental or intervention-based designs (e.g., pre-test–post-test studies). Studies conducted outside the health sector. Studies that have not examined the relationship between job stress and intention to leave the job. Studies that contain insufficient statistical data make it impossible to calculate the effect size.	6
Information sources	6	Relevant publications were identified through academic databases, including Scopus, Web of Science, PubMed, and Cochrane. Effect sizes and variances were calculated using CMA & R software. In this study, a literature search was conducted between 30 November 2025, and 20 December 2025.	5, 6
Search strategy		In this study, a literature search was conducted between 30 November 2025, and 20 December 2025 using the Scopus, WoS, PubMed and Cochrane databases. The search strategy targeted publications on job stress and turnover intention, using the following keyword combination: (“Job stress” OR “Work stress” OR “Occupational stress” OR “Workplace stress” OR “Job-related stress” OR “Work-related stress”) AND (“Turnover intention” OR “Intention to quit”). Our search strategy included the outcome terms “Turnover intention” and “Intention to quit”, but did not explicitly include other synonymous terms such as “Intention to leave” or “Intent to leave.” A total of 11,807 studies related to these two topics were identified in the Web of Science, Scopus, PubMed, and Cochrane databases, of which 6490 were classified as articles. Studies that did not directly address the relationship between job stress and intention to leave, or that were designed using qualitative methods, were eliminated at this stage. 61 studies deemed suitable for full-text review were subjected to a detailed evaluation. As a result of this evaluation, 23 studies with missing statistical data (sample size, r-value, or t/F values that could be converted) or focusing on irrelevant variables were excluded from the analysis. The remaining 38 studies that fully met the inclusion criteria were included in the quantitative synthesis stage.	7, 19
Selection process	8	The study selection process was conducted by two independent researchers. First, all records obtained from the databases were transferred to a reference management program, and duplicate records were removed. Then, during the title and abstract search phase, each record was independently evaluated by the two researchers according to predetermined inclusion and exclusion criteria. Full texts of studies deemed suitable were obtained, and full-text evaluations were again performed independently by the two researchers. When disagreements arose between the evaluators, the relevant study was discussed, and a joint decision was reached; in cases where consensus could not be reached, the opinion of a third researcher was sought. During the title–abstract search and full-text review phases, all records and reports were reviewed by at least two evaluators.	9
Data collection process	9	Data extraction from the included studies was performed independently by two researchers. During the data collection process, for each study, author(s), publication year, country, sample size, participant characteristics, measurement tools used, and statistical information showing the relationship between job stress and intention to leave (Pearson correlation coefficient [r] or statistics convertible to r-value) were recorded using a standard data extraction form (using Excel). The researchers collected the data independently, and the resulting data were then compared. Inconsistencies that arose during the data extraction process were resolved through mutual discussion and agreement. Since all necessary information was obtained from the full texts of the published articles, the study authors were not contacted for additional data requests or data verification.	9
Data items	10a	In this meta-analysis, the primary outcome variable for which data were collected is the relationship between job stress and intention to leave among healthcare workers. The Pearson correlation coefficient (r) reflecting this relationship was accepted as the primary effect size. Where the correlation coefficient was not directly reported in the studies, t, F, regression coefficients, or other appropriate statistical values were recorded to be converted to effect size. All appropriate outcomes showing a relationship between job stress and intention to leave in the included studies were examined, and statistical data that allowed for the calculation of effect size were collected. Since this relationship was reported with a single correlation coefficient in the vast majority of studies, no additional decision-making process regarding outcome selection was required.	1, 5, 6, 7, 8
	10b	In addition to the outcome variable, data on various study and sample characteristics were collected from the included studies. For each study, the author name, publication year, country where the study was conducted, healthcare professional group (nurse, doctor, therapist, or other healthcare personnel), sample size, research design, work stress and intention to leave measures used, and statistical information enabling effect size calculation were recorded. Additionally, demographic information and information on participants’ work environment were collected where available for use in moderator analyses. In the data extraction process, only information explicitly reported in published studies was used. In cases of missing or unclear information, study authors were not contacted. No values were assigned to variables not included in the reports, and the relevant information was considered “unreported”. Studies were excluded from the meta-analysis if the necessary statistical information for effect size calculation was not explicitly presented.	1, 5, 6, 7, 8
Study risk of bias assessment	11	Various methods are used to assess the quality and risk of bias in studies. In this study, since all 38 articles included were cross-sectional studies, the AXIS assessment tool was used to evaluate study quality and risk of bias. The Cross-Sectional Study Assessment Tool (AXIS tool) is specifically designed for cross-sectional research and is one of the recommended instruments for this study design [82]. To assess the quality and risk of bias in cross-sectional studies, the AXIS evaluation tool was used. The AXIS evaluation tool consists of 20 questions, with a total score ranging from 0 to 20. An increase in the total score indicates improved study quality [73]. Furthermore, the risk of bias was reviewed by two independent researchers and assessed at the outcome level, as in the study by Sayık et al. [74]. In this study, the “funnel plot” was initially used to assess the potential for publication bias. Visual inspection of the funnel plot (Figure 2) indicated that publication bias was not sufficient to threaten the validity of the meta-analytic findings	9, 10, 11
Effect measures	12	In this meta-analysis, the Pearson correlation coefficient (r) between job stress and intention to leave was used as a measure of effect size. The overall effect size obtained by synthesizing independent studies examining the relationship between job stress experienced by healthcare workers and their intention to leave their jobs demonstrates a positive, moderate-to-high correlation between these two variables [(r = 0.441, 95% CI [0.389, 0.490], *p* < 0.0001)].	10, 11, 12
Synthesis methods	13a	The suitability of studies for meta-analytic synthesis was evaluated according to predetermined inclusion and exclusion criteria. First, the basic characteristics of the included studies (year of publication, country, healthcare worker group, sample size, measurement tools used, and reported statistical data) were summarized in a table. Then, each study was evaluated in terms of whether it examined the relationship between job stress and intention to leave the job and whether it contained statistical information that allowed for the calculation of effect size. Only studies conducted in a sample of healthcare workers, reporting the relationship between job stress and intention to leave the job, and providing statistical data that could be converted to a Pearson correlation coefficient (r) or r-value were included in the meta-analytic synthesis. Studies that did not allow for the calculation of effect size were excluded from the quantitative synthesis.	1, 6
	13b	In the process of preparing the data for meta-analytic synthesis, statistical information showing the relationship between job stress and intention to leave the job was first extracted from the included studies. In studies where the Pearson correlation coefficient (r) was directly reported, these values were used.	1, 6
	13c	The characteristics and findings of individual studies are presented in summary tables. These tables report author information, publication year, correlation coefficient, country, sample size, relevance of the studies to COVID-19, and statistical data used to calculate effect size for each study. The meta-analytic synthesis results are presented visually using forest plots showing the effect size and 95% confidence interval for each study. Forest plots show the effect sizes of individual studies, their weights, and the combined effect size. In addition, heterogeneity statistics between studies and overall effect size results are reported numerically. Funnel plots were used where appropriate to assess publication bias, and the relevant statistical test results are reported.	8, 1, 11, 12
	13d	The results of the studies were synthesized using the meta-analysis method. Pearson correlation coefficients (r) were used as effect sizes. Statistical heterogeneity between studies was evaluated using Cochran’s Q test and the I^2^ statistic. A significant Q test and a high I^2^ value were interpreted as indicating the presence of heterogeneity between studies. I^2^ values were generally considered to be low (25%), medium (50%), and high (75%) heterogeneity levels. Meta-analyses were performed using Comprehensive Meta-Analysis (CMA) & R softwares. Combined effect sizes, 95% confidence intervals, and heterogeneity statistics were calculated and reported using this software.	12, 1, 5
	13e	Subgroup analysis and meta-regression were used to investigate possible reasons for heterogeneity among the studies. Subgroup analyses were conducted by occupational group, COVID-19 period, and geographic region, while meta-regression tested moderating effects of continuous variables. Meta-regression analyses were used to test the moderating roles of continuous variables on effect size.	1, 2, 13, 15, 5, 16
	13f	No additional sensitivity analysis was performed to evaluate the robustness of the results synthesized in this study.	1 (All study)
Reporting bias assessment	14	Publication bias analyses were performed to assess the risk of bias arising from incomplete results in the syntheses. A funnel plot was used to visually evaluate publication bias. Asymmetry in the funnel plot was interpreted as an indicator of possible publication bias or small sample size effects.	10, 11
Certainty assessment	15	In this study, a structured approach such as GRADE was not used to assess the certainty or confidence level of the evidence. The interpretation of the evidence considered the results of effect sizes, confidence intervals, levels of heterogeneity, and publication bias analyses.	1 (All study)
**RESULTS**			
Study selection	16a	The search strategy targeted publications on job stress and turnover intention, using the following keyword combination: (“Job stress” OR “Work stress” OR “Occupational stress” OR “Workplace stress” OR “Job-related stress” OR “Work-related stress”) AND (“Turnover intention” OR “Intention to quit”). A total of 11,807 studies related to these two topics were identified in the Web of Science, Scopus, PubMed, and Cochrane databases, of which 6490 were classified as articles. Studies that did not directly address the relationship between job stress and intention to leave, or that were designed using qualitative methods, were eliminated at this stage. 61 studies deemed suitable for full-text review were subjected to a detailed evaluation. As a result of this evaluation, 23 studies with missing statistical data (sample size, r-value, or t/F values that could be converted) or focusing on irrelevant variables were excluded from the analysis. The remaining 38 studies that fully met the inclusion criteria were included in the quantitative synthesis stage.	5, 6, 7
	16b	As a result of this evaluation, 23 studies with missing statistical data (sample size, r-value, or t/F values that could be converted) or focusing on irrelevant variables were excluded from the analysis.	7
Study characteristics	17	A total of 38 articles were included in the meta-analysis. The included studies were published between 2009 and 2025, with a relatively balanced distribution across years. Among the 38 studies, samples consisted of nurses (*n* = 21), mixed healthcare personnel (*n* = 9), physicians *(n* = 5), and therapists (*n* = 3). Twelve of these studies were conducted in the Middle East region, 22 in East Asia, and 4 in the West/Other region. In this study, the articles included were not selected based on their country of origin; all articles found in relevant databases and meeting the necessary inclusion criteria were included. Detailed characteristics of the included studies are presented in Table 1 in the article.	8
Risk of bias in studies	18	The AXIS assessment tool was used to evaluate quality and risk of bias in cross-sectional studies. The AXIS assessment tool consists of 20 questions with a total score ranging from 0 to 20. An increase in the total score indicates improved study quality [73]. Furthermore, the risk of bias was examined by two independent researchers and assessed at the outcome level, as in the study by Sayık et al. [74]. Again, disagreements between evaluators were resolved through discussion or with the participation of a third evaluator. The study shows that all 38 studies included in the research generally had high quality scores.	9, 10
Results of individual studies	19	For each included study, sample size, effect size (Pearson correlation coefficient, r), and 95% confidence interval were reported. Individual study results are presented in tabular form and visualized using a forest plot showing the effect size, confidence interval, and weight of each study in the meta-analysis. This allows for the assessment of the direction, magnitude, and consistency of effect sizes among studies. This can be seen in detail in Figure 2 included in the study.	11
Results of syntheses	20a	The studies included in the meta-analysis were conducted on a sample of healthcare workers, including nurses, doctors, and other healthcare professionals. The studies were carried out in different countries, and sample sizes ranged from 50 to 10,457. All included studies had a cross-sectional research design and examined the relationship between job stress and intention to leave the job. Pearson correlation coefficients or statistically convertible data were used to calculate effect sizes. The methodological quality of the studies was assessed using the AXIS evaluation tool. The evaluation results showed that the studies generally had acceptable methodological quality.	1
	20b	The results of the 38 studies included in the meta-analysis were synthesized using the random effects model. Findings revealed a moderate-to-high, positive, and statistically significant relationship (r = 0.441; 95% CI [0.389, 0.490]) between job stress and turnover intention among healthcare workers. Moreover, although between-study heterogeneity was substantial (I^2^ = 95.5%), the direction and statistical significance of the association remained consistent across different countries, healthcare systems, and professional groups. Given the substantial heterogeneity among the included studies (Q = 819.85, *p* < 0.001; I^2^ = 95.5%), a random-effects model was applied in all analyses. Information regarding the studies examining the association of job stress on turnover intention in healthcare institutions.	1, 8
	20c	Although extensive subgroup and meta-regression analyses were conducted to explore potential sources of heterogeneity, a high level of between-study heterogeneity remained (I^2^ = 95.5%). This suggests that additional unmeasured contextual or methodological factors—such as institutional characteristics, health system structures, workload intensity, or variations in measurement instruments—may have influenced the observed effect sizes and could not be fully captured in the present analyses.	1, 8
	20d	In this study, a sensitivity analysis was not conducted to evaluate the robustness of the meta-analysis results.	1
Reporting biases	21	In this study, the “funnel plot” was initially used to assess the potential for publication bias. Visual inspection of the funnel plot (Figure 3) indicated that publication bias was not sufficient to threaten the validity of the meta-analytic findings. The results of the funnel plot are presented in Figure 3.	11
Certainty of evidence	22	The certainty or confidence level of the evidence regarding the results evaluated in this study was not assessed using GRADE or a similar structured methodology.	1
**DISCUSSION**			
Discussion	23a	Based on predefined inclusion criteria, 38 independent studies were included. Findings revealed a moderate-to-high, positive, and statistically significant relationship (r = 0.441; 95% CI [0.389, 0.490]) between job stress and turnover intention among healthcare workers. Moreover, although between-study heterogeneity was substantial (I^2^ = 95.5%), the direction and statistical significance of the association remained consistent across different countries, healthcare systems, and professional groups.	1
	23b	Some limitations must be considered when interpreting the studies included in this review. First, since almost all of the included studies have a cross-sectional research design, it is not possible to make definitive conclusions about the causal aspect of the relationship between job stress and intention to leave the job. Although extensive subgroup and meta-regression analyses were conducted to explore potential sources of heterogeneity, a high level of between-study heterogeneity remained (I^2^ = 95.5%). This suggests that additional unmeasured contextual or methodological factors—such as institutional characteristics, health system structures, workload intensity, or variations in measurement instruments—may have influenced the observed effect sizes and could not be fully captured in the present analyses. Second, all included studies employed cross-sectional and observational survey designs. Therefore, the findings of this meta-analysis should be interpreted as associational rather than causal in nature. Although a robust and statistically significant relationship between job stress and turnover intention was identified, the present study does not permit causal inferences regarding the directionality of this relationship or the effectiveness of specific organizational interventions.	18
	23c	In this study, the literature search was limited to the Scopus, Web of Science, PubMed, and Cochrane databases. Therefore, it is possible that relevant studies indexed in other databases or in the grey literature were overlooked. Secondly, only studies providing sufficient statistical data to allow for effect size calculation were included in the meta-analysis. Exclusion of studies that did not report the necessary statistical information may have limited the scope of the findings.	1, 18, 19
	23d	Given the limited number of meta-analyses in healthcare management and human resources literature, this study provides a meaningful contribution by clarifying the relationships of job stress with turnover intention and highlighting the importance of addressing stress in healthcare institutions. Employee engagement should be enhanced through reward and recognition systems; corporate loyalty programs and work–life balance initiatives should be implemented. The Ministry of Health and relevant institutions should establish regulations to combat job stress (e.g., limits on shift durations, rest rights). Institutional systems to monitor stress levels should be developed in public hospitals, and these data should be used in managerial decision-making.	1, 19
**OTHER INFORMATION**			
Registration and protocol	24a	The study was registered in the International Prospective Register of Systematic Reviews (PROSPERO; ID: CRD42024581906).	1
	24b	The protocol for this systematic review and meta-analysis study has been prepared in advance and registered in the PROSPERO database (Registration No: CRD42024581906). The protocol is accessible via the PROSPERO registration system.	1
	24c	Following the PROSPERO registration, the research team was updated. Researchers who made significant scientific contributions in later stages of the study were added to the author list, taking into account international authorship criteria. This change only pertained to authorship information and did not affect the study protocol, research questions, eligibility criteria, or analysis methods.	PROSPERO ID: Registration No: CRD42024581906.
Support	25	No financial support, grants, or external funding was received for this study. The study was conducted using the authors’ own academic efforts and institutional resources.	20
Competing interests	26	The authors declare no conflicts of interest regarding the publication of this article.	20
Availability of data, code and other materials	27	All data generated or analyzed during this study are included in this published article.	1, 20
